# Production of spiculisporic acid by *Talaromyces trachyspermus* in fed-batch bioreactor culture

**DOI:** 10.1186/s40643-021-00414-1

**Published:** 2021-07-10

**Authors:** Maki Moriwaki-Takano, Chikako Asada, Yoshitosi Nakamura

**Affiliations:** 1 Graduate School of Science and Engineering, University of Toyama, 3190 Gofuku, Toyama, 930-8555 Japan; 2 Department of Bioscience and Bioindustry, Tokushima University, 2-1 Minamijosanjima-cho, Tokushima, 770-8513 Japan

**Keywords:** Biosurfactant, Spiculisporic acid, *Talaromyces trachyspermus*, Fed-batch culture, Bioreactor

## Abstract

Spiculisporic acid (SA) is a fatty acid-type biosurfactant with one lactone ring and two carboxyl groups. It has been used in metal removers and cosmetics, because of its low propensity to cause irritation to the skin, its anti-bacterial properties, and high surface activity. In the present study, we report an effective method for producing SA by selecting a high-producing strain and investigating the effective medium components, conditions, and environments for its culture. Among the 11 kinds of *Talaromyces* species, *T. trachyspermus* NBRC 32238 showed the highest production of a crystalline substance, which was determined to be SA using NMR. The strain was able to produce SA under acidic conditions from hexoses, pentoses, and disaccharides, with glucose and sucrose serving as the most appropriate substrates. Investigation of nitrogen sources and trace metal ions revealed meat extract and FeCl_3_ as components that promoted SA production. Upon comparing the two types of cultures with glucose in a baffle flask or aeration bioreactor, SA production was found to be slightly higher in the flask than in the reactor. In the bioreactor culture, sucrose was found to be an appropriate substrate for SA production, as compared to glucose, because with sucrose, the lag time until the start of SA production was shortened. Finally, fed-batch culture with sucrose resulted in 60 g/L of SA, with a total yield of 0.22 g SA/g sucrose and a productivity of 6.6 g/L/day.

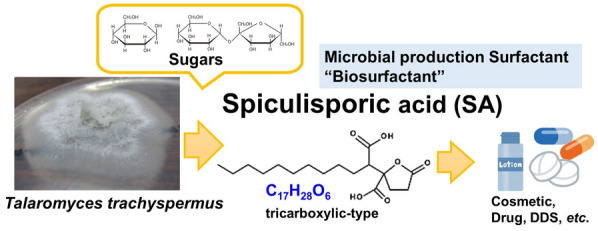

## Introduction

Surfactants are among the most versatile chemicals used in a variety of industries, including detergents, paints, paper products, pharmaceuticals, cosmetics, petroleum, food, and water treatment (Elazzazy et al. 2015; Mahamallik and Pal 2017; Varjani and Upasani 2017). At present, domestic annual surfactant production in Japan is more than 1.1 million tons, a number that is increasing year by year (Year Book of Current Production Statics, Chemical Industry 2019). Commercially available surfactants can be synthesized from petrochemicals, animal fats, plants, and microorganisms; however, most surfactant production relies on petrochemicals (Rufino et al. 2014; Melo et al. 2015; De Almeida et al. 2016). An increase in the amount of oil chemicals causes two phenomena: an increase in environmental pollution, which affects public health, and depletion of oil production within the next few decades (Frumkin et al. 2009). Sustainable social growth demands the development of new strategies to replace fossil fuel products with renewable and biodegradable materials. For the same reason, surfactants made from natural resources using biotechnology, known as biosurfactants, are being focused on to replace synthetics derived from petroleum. Biosurfactants produced by the metabolism of organisms are characterized by low toxicity, bioavailability, biodegradability, high effervescence, and environmental friendliness, and can be widely used in foods, pharmaceuticals, cosmetics, etc. (Ahmadi-Ashtiani et al. 2020). These have been developed and used as multifunctional agents, such as stabilizers, wetting agents, anti-bacterial agents, humectants, emulsifiers, and anti-adhesion agents (Banat et al. 2000; Vijayakumar and Saravanan 2015). However, the mass production of many biosurfactants is difficult and expensive because of the low productivity of microorganisms and low availability of raw materials (Santos et al. 2016).

These biosurfactants are biosynthesized primarily as secondary metabolites and play an important role in microbial growth and localization. Based on the chemical structure of the hydrophobic component, biosurfactants are classified into four categories: (1) glycolipid-type, (2) fatty acid-type, (3) lipopeptide-type, and (4) polymer-type (Raffa et al. 2015; Vijayakumar and Saravanan 2015). Glycolipid-types include rhamnolipids, sophorolipids, and mannosylerythritol lipids. For example, rhamnolipids are produced by *Pseudomonas* sp., and surfactin of the cyclic lipopeptide is produced by *Bacillus subtilis* (Soberón-Chávez et al. 2005; Chen et al. 2015).

Spiculisporic acid (4,5-dicarboxy-4-pentadecanolide, SA) is a fatty acid-type biosurfactant (Ishigami et al. 2013). The tricarboxylic-type surfactant structure has recently received attention as a commercially available biosurfactant and a new biomaterial owing to its safety for human health and the environment. It contains 2–3 carboxylic acids and the physiological properties of a surfactant. SA was discovered as a metabolite from *Penicillium spiculisporum*, which was isolated from the precipitate crystallized in the acidified culture broth of the fungus (Clutterbuc et al. 1931). Isotope uptake studies and enzyme purification showed that SA biosynthesis involves the condensation of lauroyl-CoA and 2-oxoglutarate (Måhlén 1971, 1973). The reaction is catalyzed by decylhomocitrate synthase. Enantioselective synthesis of SA has also been reported (Brandänge et al. 1984; Brown et al. 2003). SA is used in commercial applications to prepare new emulsion-type organogels, superfine microcapsules (vesicles or liposomes), heavy metal sequestrants, and in many other applications in specialty chemicals and biotechnology (Hong et al. 1998; Pekdemir et al. 2000). The raw material has low surface activity, which increases in the derivatives (Yamazaki et al. 1983; Choi et al. 1993). The derivatives promote transdermal absorption of the moisturizer. Due to their physiological properties, these have potential use as new bioactive substances in the pharmaceutical, cosmetic, agricultural, and biotechnology industries.

*Talaromyces trachyspermus* is the perfect state teleomorph of *P. spiculisporum.* The genus *Talaromyces* was first introduced by Benjamin in 1955 as teleomorphs of the *Penicillium* species, with woven hyphae and soft cleistothecia ascomata (Benjamin 1955). It was redefined by Stolk and Samson in 1971 (Stolk and Samson 1971; Fennell 1973). *T. trachyspermus* are characterized by conspicuously spindle-shaped, elliptical ascospores, and a clear ascotic covering. *T. trachyspermus* has been found to produce a fatty acid surfactant, trachyspic acid [2-(carboxymethyl)-8-nonyl-9-oxo-1,6-dioxaspiro[4.4]non-7-ene-2,3-dicarboxylic acid], which inhibits heparanase (Shiozawa et al. 1995). Since the structure of the product is similar to that of SA produced by *P. spiculisporum*, the strain was predicted to produce SA. Its derivatives are found in *T. trachyspermus* and *Aspergillus candidus* cultures (Wang et al. 2012, 2015; Kumla et al. 2014). While various reports have shown SA production and characterization, its industrial production is limited. It is manufactured in Japan by only a single source, Iwata Chemical Industry Co. Ltd., which has established a manufacturing method for it. Annual production is estimated to be 4–5 tons. Mass production and scale-up have inevitable problems in trying to achieve low production costs, low environmental pollution, and advanced innovation in the industrial production of fermented products (Chakravarty et al. 2017). Fed-batch (semi-batch) operation is a practical cultivation technology to overcome the various problems that are faced when performing batch and continuous operation (El Moslamy 2019).

In this study, the effective production of SA by *T. trachyspermus* was investigated by changing the medium composition and culture conditions. In the shake flask-scale step, the microbial response to medium components (carbon sources, nitrogen sources, and trace metals) and pH conditions was assessed for SA production. Scaled-up cultivation was attempted in an aeration and stirring bioreactor, to achieve high SA productivity. The utility of two types of culture was evaluated: normal batch culture and fed-batch culture, in which two substrates of glucose or sucrose solution were added to the culture medium.

## Materials and methods

### Microorganisms

*Talaromyces trachyspermus* NBRC 6440, 8890, 9861, 31360, 31757, 31907, 32238, 106931, and 106932, *T. assiutensis* NBRC 30691, and *T. assiutensis* NBRC 31750 were purchased from the Biological Resource Center of the National Institute of Technology and Evaluation (Japan). These fungi were maintained on potato dextrose agar (PDA; Difco Laboratories, Detroit, MI, USA) slants at 2–8 °C. A fungal pre-culture was prepared by cultivating the fungus on a PDA plate at 28 °C for 7 days.

### Basic medium

Fungi were mainly cultivated in basic medium containing the following (per L): 0.5 g KH_2_PO_4_, 0.5 g MgSO_4_·7H_2_O, 0.0915 g FeSO_4_·7H_2_O, 0.8 g (NH_4_)_2_SO_4_, 1.0 g corn steep liquor (CSL, Sigma-Aldrich, St. Louis, MO, USA) and 100 g glucose. Glucose was mainly used as a carbon source. All the reagents used, except CSL, were purchased from FUJIFILM Wako Pure Chemical Co. (Osaka, Japan). The initial pH of the medium was adjusted to 2–4 using HCl aq. before sterilization. The medium was autoclaved at 121 °C for 15 min. The carbon sources used in this study were hexoses (glucose, galactose, and fructose), pentoses (xylose and arabinose), and disaccharides (sucrose and lactose) at an initial concentration of 100 g/L. The nitrogen sources of the natural extracts were casamino acid, casein (Sigma-Aldrich), malt extract (Difco Laboratories), meat extract (Kyokuto Pharmaceutical Industrial Co. Ltd., Tokyo, Japan), and Hipolypepton (Nihon Pharmaceutical Co. Ltd., Tokyo, Japan). The salts used as nitrogen source were KNO_3_, NaNO_3_, NH_4_NO_3_, (NH_4_)_2_SO_4_, and urea (FUJIFILM Wako Pure Chemical Co.), which were added to the basic medium at a concentration of 5 g/L. The mineral components used were CaCl_2_, CoCl_2_, CuSO_4_, FeCl_2_, FeCl_3_, FeSO_4_, Fe_2_(SO_4_)_3_, MnSO_4_, NiCl_2_, ZnCl_2_, and ZnSO_4_, which were added to the basic medium at a concentration of 5 mg/L.

### Batch flask culture

Cultivations using 13 types of filamentous fungi were performed in an Erlenmeyer flask, as follows: hyphae piece ~ 1 cm^2^ grown on the pre-culture PDA plate was inoculated in a 500-mL Erlenmeyer flask with three baffles containing 100 mL of the medium. The flasks were cultured with shaking on a rotary shaker at 28 °C and 140 rpm for 7 days.

After cultivation, the mycelium and SA were separated from the culture solution using a filter paper (Advantec^®^ No. 131, 110 mm, ADVANTEC Toyo Kaisha, Ltd., Tokyo, Japan). The filtrate was analyzed using HPLC. The solid phase on the filter was washed several times with distilled water. After removing the water, ethanol was poured above the filter paper to dissolve the SA, followed by collection of the filtrate. The solution containing SA was evaporated at 50 °C for 24 h, and the weight of the product was measured. After washing with ethanol, the residue was dried in an oven at 90 °C for 24 h, to estimate the growth of fungi in terms of dry cell weight (DCW).

### Bioreactor culture

An aeration stirring-type bioreactor with a volume of 2 L was used (Sakura Seiki Co., Nagano, Japan). Batch cultures were performed in 1 L of the medium with glucose or sucrose in the bioreactor, which was autoclaved at 121 °C for 45 min. The cultivation was started with the mycelium of *T. trachyspermus* inoculated into the bioreactor under aseptic conditions. It was controlled at 28 °C and 700 rpm, with an aeration rate of 0.5 vvm for 11–19 days.

Fed-batch cultures were performed in the same reactor with either glucose or sucrose. During the culture, 200 mL of 500 g/L sterilized sugar solution was added from the insertion port of the bioreactor, at 6 and 11 days for glucose, and at 4 and 8 days for sucrose.

### HPLC assay

Culture broth samples filtered with filter paper were analyzed using HPLC (LC-10 system: Shimadzu Co., Kyoto, Japan) using a refraction detector (RID-10A, Shimadzu Co.) at 40 °C with 1.25 mM sulfuric acid as the mobile phase, at a flow rate of 0.6 mL/min. The column used was an IC Sep WA1 Wine Analysis Column (Transgenomic Co., NE, USA).

### NMR analysis

The refined product was analyzed using NMR, to identify the crystals secreted from *T. trachyspermus*. The recrystallized SA was analyzed using ^1^H and ^13^C-NMR (400 MHz, JNM-ECS400, JEOL Ltd., Tokyo, Japan) after evaporation of ethanol from the SA solution recovered from the culture. The solvent used was 99.5% ethanol-d6, and the inner standard was 0.005 v/v% tetramethylsilane.

## Results and discussion

### Screening for a biosurfactant-producing strain

**Fig. 1:**
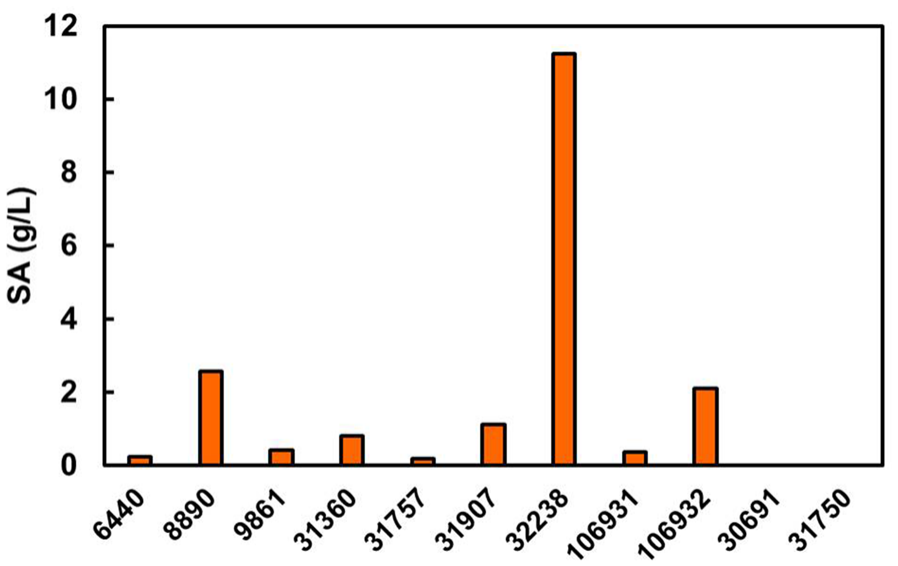
Screening for an spiculisporic acid-producing fungus from *Talaromyces* species. Cultivation was performed in a baffle flask for 7 days. The medium volume was 100 mL, containing 100 g/L glucose. The culture conditions were pH 3.0 and a temperature of 28 °C

Eleven strains of *Talaromyces* were compared for SA production, to select for the high-producing strains. These were cultured with 100 g/L glucose in a baffle flask at pH 3.0, 28 °C for 7 days. The pre-experiment showed that the strains produced SA under conditions of high substrate concentration and low pH, under adequate aeration. *T. trachyspermus* was able to produce SA, whereas *T. assiutensis* only grew (Fig. 1). SA dispersed into the culture medium in a crystalline form as soon as it was produced, because it is hardly soluble in water. The medium became cloudy in the later stage of the culture. *T. trachyspermus* NBRC 32238 produced the highest SA yield of 11.3 g/L, while the others produced a slight amount of SA, of no more than 3 g/L. The yield of NBRC 32238 (based on sugar consumption) was 0.333 g SA/g glucose, while the productivity was 0.012 g/L/day. The *T. trachyspermus* NBRC 32238 was selected as the most efficient SA-producing strain and used in the subsequent studies.

### Identification of the *T. trachyspermus* product using NMR

**Fig. 2:**
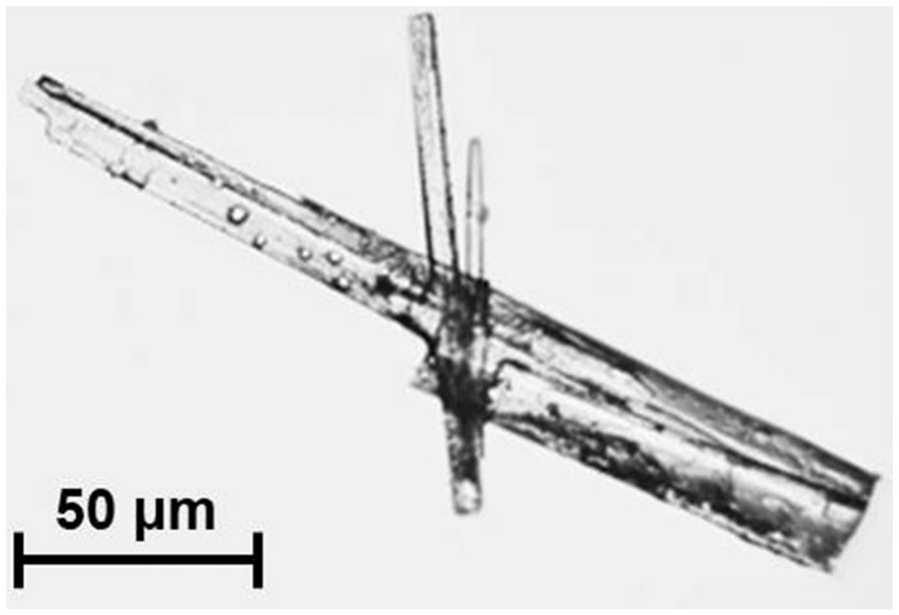
A photograph of spiculisporic acid crystals. The product was purified from *T. trachyspermus* NBRC 32238 using ethanol elution and recrystallization

**Fig. 3:**
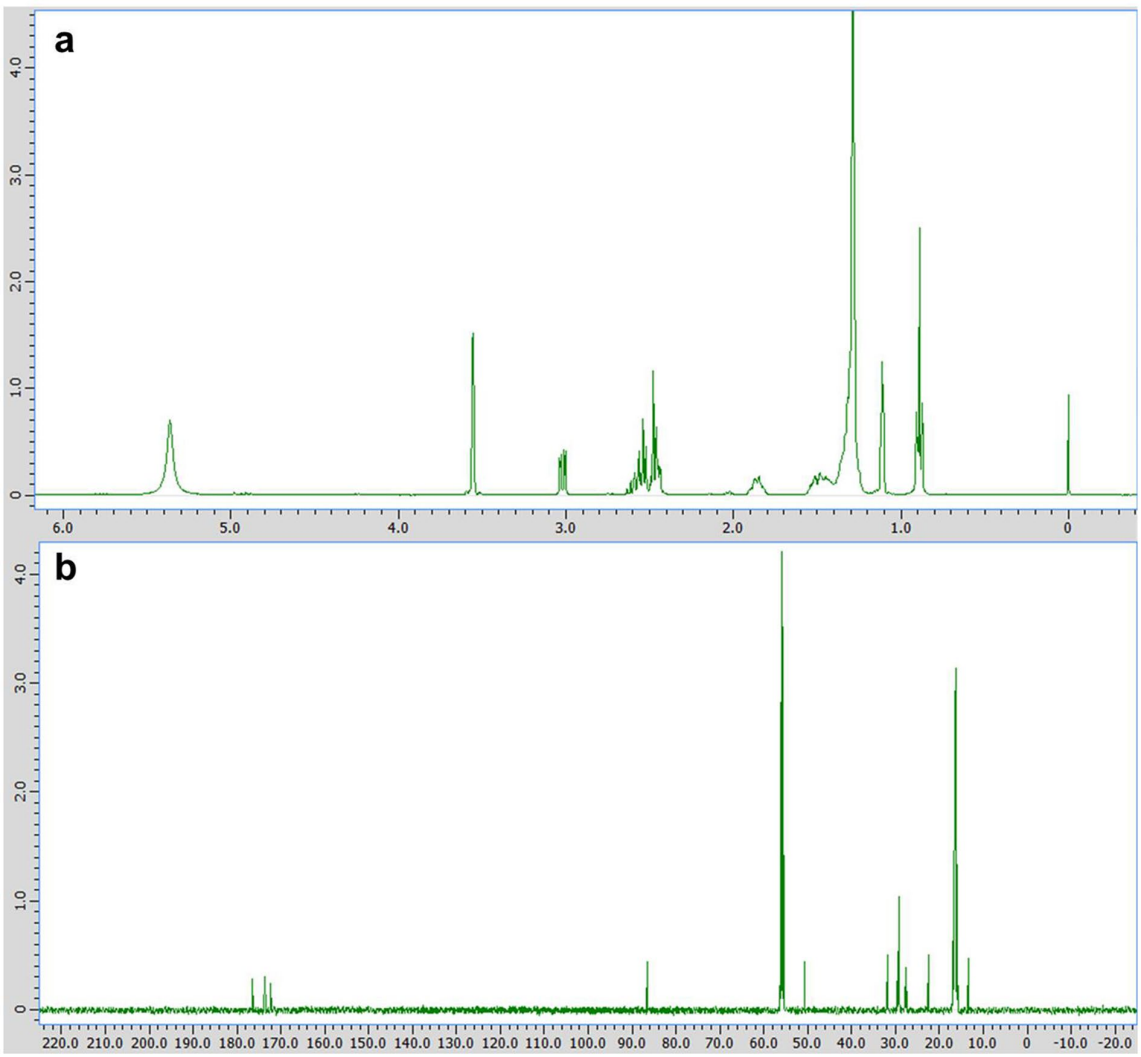
Analysis of the product from *T. trachyspermus* NBRC 32238 using NMR. Spectrum data of **a **^1^H NNR **b **^13^C NMR. The solvent used was 99.5% ethanol-d6, and the inner standard was 0.005 v/v% tetramethylsilane

**Fig. 4:**
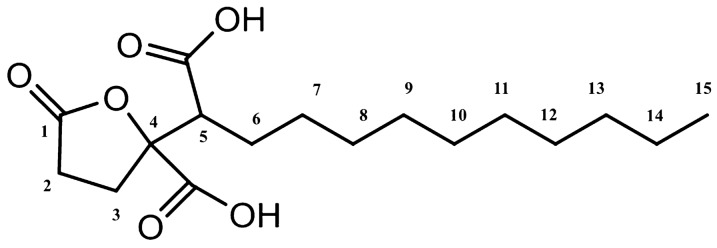
The structure of spiculisporic acid produced from *T. trachyspermus* NBRC 32238

The substance secreted from *T. trachyspermus* NBRC 32238 was isolated as white needle-like solid with extremely high solubility in ethanol and poor solubility in water. A photograph of the SA crystal is shown in Fig. 2. Its molecular formula was determined by ESI high-resolution mass spectrometry as C_17_H_28_O_6_ [*m/z* 328.1880, calculated for 328.1886] having four degrees of unsaturation. The ^1^H-NMR spectrum of the substance displayed signals characteristic for one methine [*δ*_H_ 3.01 (dd, *J* = 8.8, 2.8 Hz, H-5)], three methylenes [*δ*_H_ 2.63–2.43 (m, H_2_-2 and H_2_-3), 1.92–1.80 and 1.56–1.48 (each m, H_2_-6)], and one methyl [*δ*_H_ 0.89 (t, *J* = 5.2 Hz, H_3_-15)] in addition to other 16 aliphatic protons [*δ*_H_ 1.38–1.28 (m, H-7 ~ 14)] (Fig. 3a). These data indicate that compound has one double bond and three carbonyls, which account for four degrees of unsaturation required by the molecular formula, so it must contain a ring. ^13^C- and DEPT NMR spectra exhibited signals corresponding to 17 carbons which can be classified as three carbonyl (*δ*_C_ 176.6, 173.6, and 172.3), one quaternary carbon (*δ*_C_ 86.5), one methine (*δ*_C_ 50.7), eleven methylenes (*δ*_C_ 31.8–22.5), and one methyl (*δ*_C_ 13.4) (Fig. 3b). The partial connectivities of C2–C3 and C5–C6 were deduced from COSY and HMQC correlations. These data of the substance were in good accordance with those from the commercially available spiculisporic acid produced by *P. spiculisporum* (Table 1). From the above reasons, we determined the isolated compound is SA (Fig. 4).

**Table 1:** NMR spectral data for commercial SA from *P. spiculisporum* and *T. trachyspermus*’ product

Position	*P. spiculisporum*	*T. trachyspermus*
^1^H-NMR spectral data		
2	2.54	2.55
3	2.46	2.45
5	3.01 (dd)	3.01(dd)
6	1.87 (m), 1.48 (m)	1.86 (m), 1.48 (m)
7	1.28–1.39 (m)^a^	1.28–1.38 (m)^b^
8	1.28–1.39 (m)^a^	1.28–1.38 (m)^b^
9	1.28–1.39 (m)^a^	1.28–1.38 (m)^b^
10	1.28–1.39 (m)^a^	1.28–1.38 (m)^b^
11	1.28–1.39 (m)^a^	1.28–1.38 (m)^b^
12	1.28–1.39 (m)^a^	1.28–1.38 (m)^b^
13	1.28–1.39 (m)^a^	1.28–1.38 (m)^b^
14	1.28–1.39 (m)^a^	1.28–1.38 (m)^b^
15	0.89 (t)	0.89 (t)
^13^C-NMR spectral data		
1	176.6 (s)	176.6 (s)
2	27.6 (t)	27.6 (t)
3	29.4 (t)	29.4 (t)
4	86.5 (s)	86.3 (s)
4-COOH	173.6 (s)	173.6 (s)
5	50.7 (d)	50.7 (d)
5-COOH	172.3 (s)	172.3 (s)
6	27.8 (t)	27.8 (t)
7	27.7 (t)	27.7 (t)
8	29.4 (t)^c^	29.4 (t)^d^
9	29.2 (t)^c^	29.2 (t)^d^
10	29.2 (t)^c^	29.2 (t)^d^
11	29.4 (t)^c^	29.4 (t)^d^
12	29.5 (t)^c^	29.5 (t)^d^
13	31.8 (t)	31.8 (t)
14	22.5 (t)	22.5 (t)
15	13.4 (q)	13.4 (q)

^a, b^Overlapping signals

^c, d^Interchangeable signals

### Effect of initial pH

**Fig. 5:**
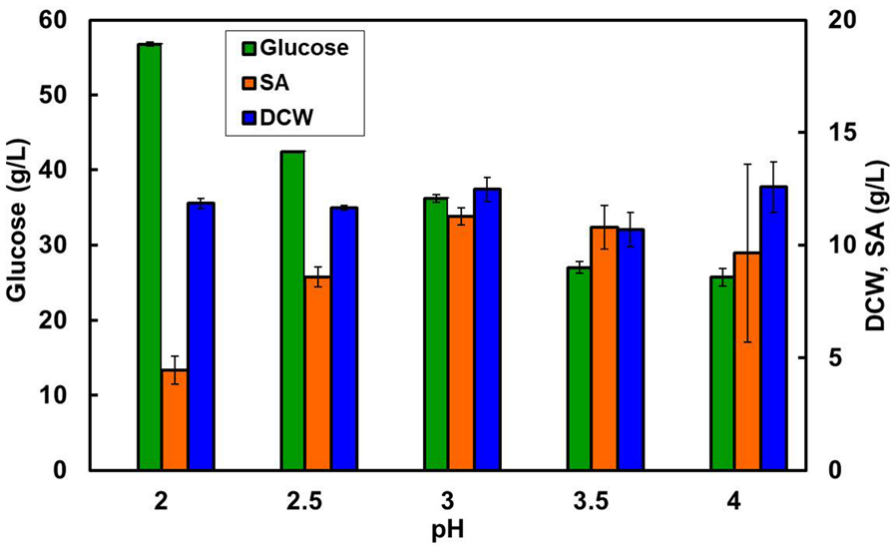
Effect of initial pH on spiculisporic acid production by *T. trachyspermus* NBRC 32238. The medium was adjusted to an initial pH of 2.0–4.0, and the strain was cultured with 100 g/L glucose at 28 °C for 7 days. Error bars indicate standard deviations from three independent experiments

The effect of initial pH on SA production was investigated because the selected strain of *T. trachyspermus* NBRC 32238 produced the substance in a low pH medium. The medium was prepared at an acidic pH of 2.0–4.0, and the strain was cultured in the medium with 100 g/L glucose at 28 °C for 7 days. Glucose consumption by the strain increased with increasing pH up to a pH of 4.0 (Fig. 5). When the initial pH was 3.0, a maximum SA amount of 11.2 g/L was produced, without linking the glucose assimilation behavior. Low pH operation can avoid contamination problems in a scale-up culture during on-site manufacturing. The strain produced the crystalline form SA at acidic pH. This phenomenon protects its own cell from the adverse effects of SA as a surfactant, because the solubility of this substance increases with environmental pH. Moreover, the salt changes the state of molecular aggregation in response to environmental pH: vesicles are formed at pH in the range of approximately 5.8–6.2, lipid particles at pH in the range of 6.3–6.6, and micelles at pH 6.8 or higher (Yamazaki et al. 1983; Ishigami et al. 2000; Kunieda and Sakamoto 2010).

### Substrate specificity

SA production by *T. trachyspermus* NBRC 32238 upon using eight types of sugars (glucose, sucrose, galactose, arabinose, xylose, lactose, fructose, and maltose) as carbon sources was assessed. It was cultivated for 7 days in basic medium containing 100 g/L of each sugar.

**Fig. 6:**
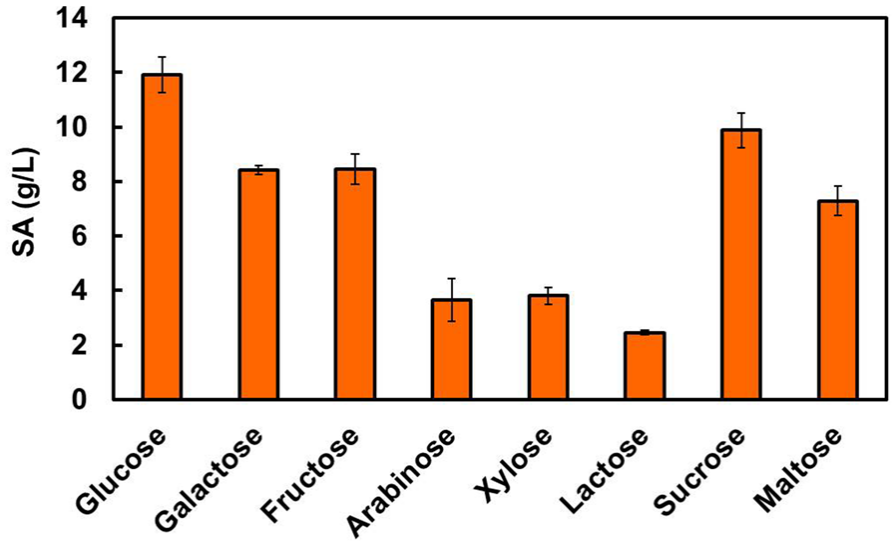
Effect of carbon sources on spiculisporic acid production by T. trachyspermus NBRC 32238. The initial substrate concentration was 100 g/L. Cultivation was carried out at 28 °C for 7 days. Error bars indicate standard deviations from three independent experiments

The strain assimilated and metabolized various carbohydrates (hexoses, pentoses, and disaccharides) (Fig. 6). SA was produced from all the sugars at concentrations in the range of 2.5–11.9 g/L. The highest SA yield of 11.9 g/L was produced upon use of glucose; the yield and productivity were 0.14 g SA/g glucose and 1.7 g/L/day based on sugar consumption, respectively. Sucrose was the most consumed carbon source and had the largest DCW, with similar SA production as the glucose culture. The strain secretes acid-resistant invertase to decompose sucrose into glucose and fructose.

Sugars, as energy sources, are involved in substance conversion along metabolic pathways for cell growth. Adequate substrates are essential for both fatty acid synthesis and promotion of the TCA cycle, since SA biosynthesis is a condensation reaction between lauroyl-CoA and 2-oxoglutarate.

### Selection of nitrogen sources

**Fig. 7:**
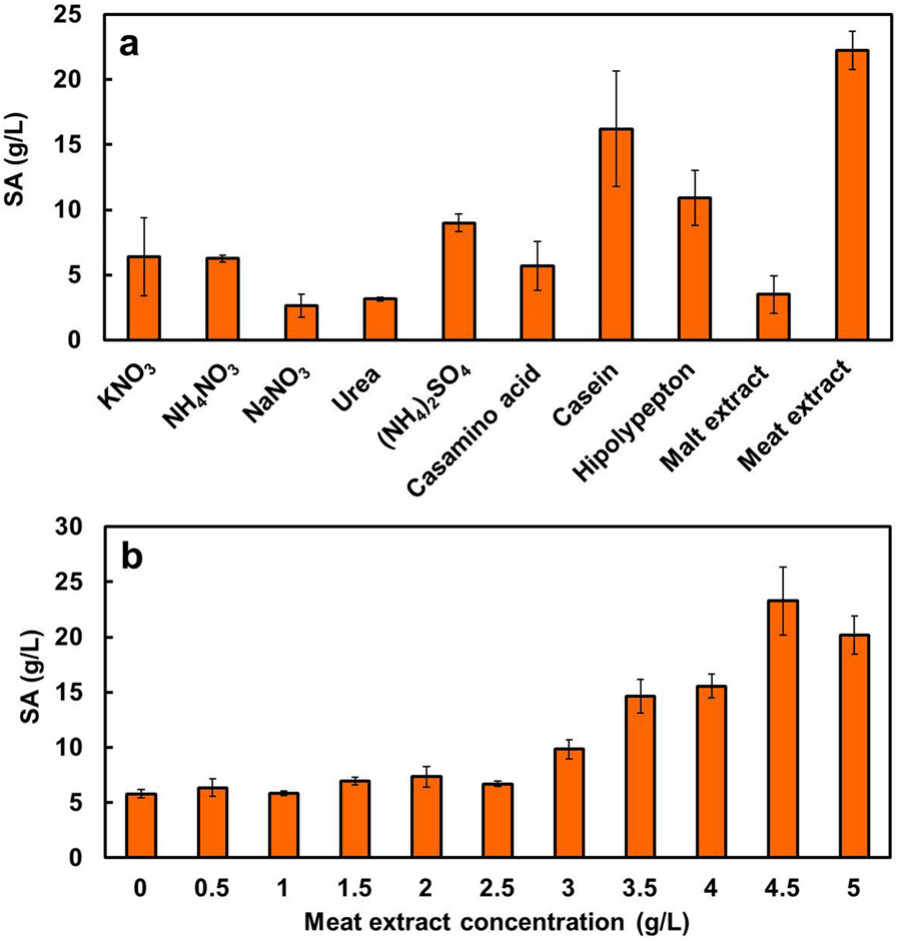
Effect of nitrogen source on spiculisporic acid production by *T*. *trachyspermus* NBRC 32238. **a** Comparison of spiculisporic acid production with various nitrogen sources. **b** Spiculisporic acid production with meat extract at concentrations of 0–5 g/L. Cultivation was carried out with 100 g/L glucose at 28 °C for 7 day. Error bars indicate standard deviations from three independent experiments

The basic medium culture containing (NH_4_)_2_SO_4_ and CSL resulted in a yield of at most 2.2 g/L of SA, as described in the previous section. Nitrogen sources affect the production of many materials as substrates of enzymes and substances involved in the different processes in microorganism cells. SA production was evaluated upon using 10 alternate nitrogen sources for (NH_4_)_2_SO_4_ in the medium. *T. trachyspermus* NBRC 32238 was cultured for 7 days with these nitrogen sources (5 g/L) in a baffle flask containing 100 g/L glucose. The strain grew and produced SA with each nitrogen source (Fig. 7a). When inorganic sources were used, glucose consumption was poor and SA production was approximately 0.5–15.0 g/L. Natural nitrogen sources improved glucose consumption and led to relatively high DCW and SA amounts. Meat extract induced the highest SA production of 22.2 g/L. It is essential to determine which components of the meat extract affect SA synthesis; however, this is challenging since the components in the extract are private.

Different meat extract concentrations (0–5 g/L) were investigated for SA production in basic medium with 100 g/L glucose at 28 °C for 7 days. SA production increased with increasing meat extract up to 4.5 g/L and reached a highest value of 23.3 g/L (Fig. 7b). The SA yield and productivity were 0.29 g SA/g glucose and 3.3 g/L/day, respectively.

The amount of the target substance depends on the type of nitrogen source to be added or the product lot. This induces a large change in production, or, in some cases, no production of the target substance. Since the meat extract used in this study was made from the meat of fishes and livestock, it contains several components that to be carbon sources and growth stimulate compounds. Though these enhanced the growth and SA production of the strain, specific components that affect the strain are unclear because details in the meat extract are private. SA synthesis requires lipid production, the formation of 2-oxoglutarate in the TCA cycle, and expression of enzymes involved in these formations because it is produced by a condensation reaction with lipids and organic acids. The formation of these enzymes necessitates the use of appropriate nitrogen sources and the meat extract also affects these reactions.

### Effect of trace metal ions

The effects of metal ions involved in SA production were examined to improve the production efficiency. Eleven types of metal salts were added to the basal medium at a concentration of 5 mg/L, and *T. trachyspermus* NBRC 32238 was cultured with 100 g/L glucose for 7 days.

**Fig. 8:**
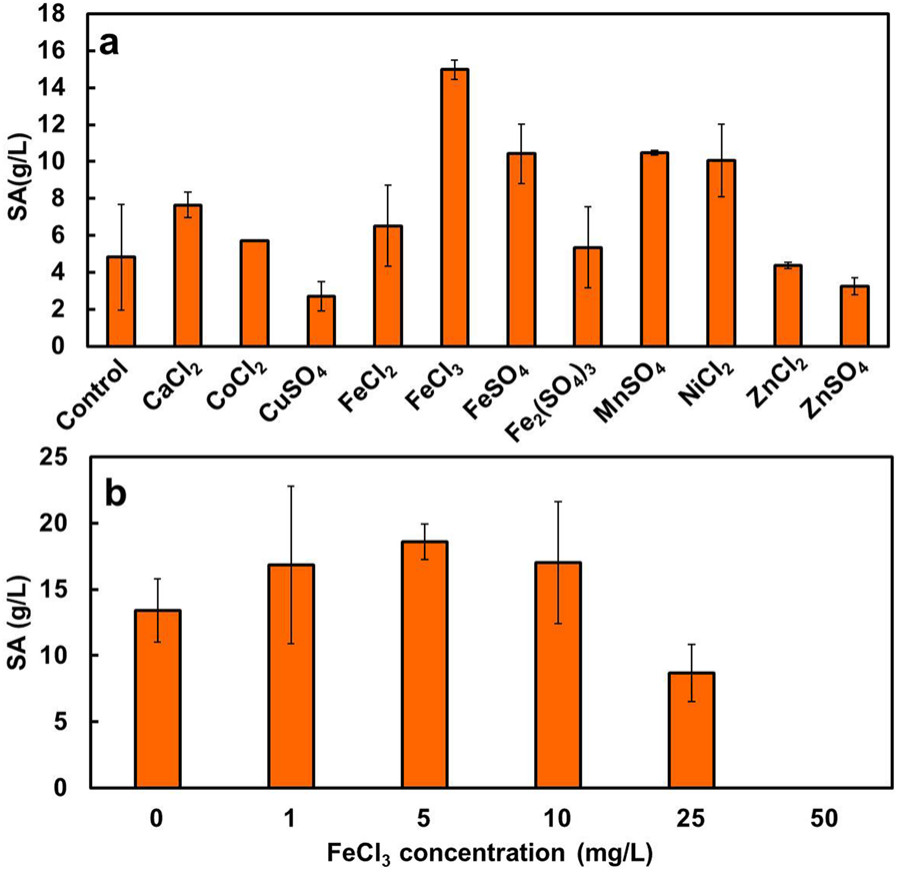
Effect of trace metal ions on spiculisporic acid production by *T. trachyspermus* NBRC 32238.** a** Comparison of spiculisporic acid production with various metal compounds. **b** Spiculisporic acid production with FeCl_3_ at concentrations of 0–50 g/L. Cultivation was carried out with 100 g/L glucose at 28 °C for 7 days. Error bars indicate standard deviations from three independent experiments

The strain produced 5.2 g/L of SA in the control case without the metal salts (Fig. 8a). Culture with the addition of metal salts such as FeSO_4_, Fe_2_(SO_4_)_3_, FeCl_3_, MnSO_4_, and NiCl_2_ promoted SA production to 2–3 times higher than that without addition. FeCl_3_ increased the glucose consumption to tenfold, as compared to the control culture, whereas the DCW was not different (data not shown). This resulted in the induction of the highest concentration of SA (15.0 g/L). The addition of iron-containing salt to the medium significantly affected the glucose consumption and SA production. The trivalent chloride iron, especially, plays a positive role in the metabolism involved in SA production in *T. trachyspermus* because only FeCl_3_ promoted both glycolysis and SA production, while FeCl_2_, FeSO_4_, and Fe_2_(SO_4_)_3_ did not.

The effects of addition of different concentrations of FeCl_3_ to the medium (0–50 mg/L) were investigated. Cultivation was performed under the same conditions as described above. The produced SA increased with increasing concentration of FeCl_3_, from 0 to 5 mg/L, and then decreased upon further addition (Fig. 8b). The strain was able to grow with 50 mg/L of FeCl_3_, but SA was not produced at this concentration. The highest amount of SA (18.6 g/L) was produced by the strain at 5 mg/L of FeCl_3_.

Trace mineral components are required in the culture medium for many physiological activities in the production of useful substances by microorganisms, for example, as an enzyme cofactor that acts on sugar metabolism, substance production, substance synthesis, and cell retention. Iron ions play an important role in energy acquisition by repeated oxidation and reduction on the electron transport chain in the inner mitochondrial membrane downstream of the TCA cycle. The trivalent chloride iron has a positive effect on the SA-producing strain. FeCl_3_ enhances the expression level of related enzymes in SA production, to increase the accumulation of fatty acids-CoA and 2-oxoglutarate generated in the TCA cycle and downstream.

### Batch culture in flask or bioreactor

**Fig. 9:**
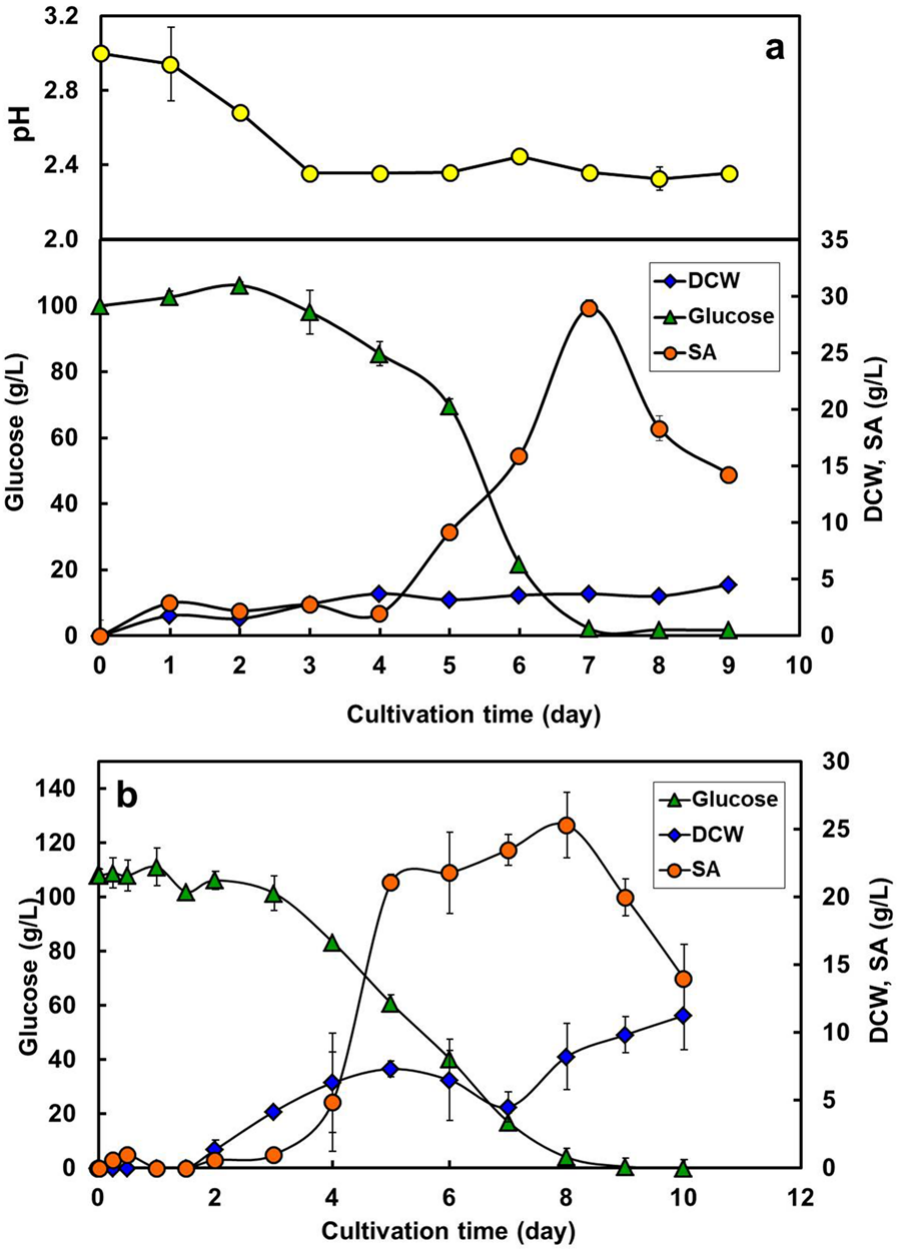
Batch fermentation profiles for spiculisporic acid production with different culture equipment. **a** Flask culture with three baffles containing 100 mL medium with 100 g/L glucose at 28 °C for 9 days. **b** Bioreactor culture by an aeration stirring-type reactor containing 1 L medium controlled at 28 °C and 700 rpm with an aeration rate of 0.5 vvm for 10 days. The cultivation was carried out with 100 g/L glucose, 4.5 g/L meat extract, and 5.0 g/L FeCl_3_. Error bars indicate standard deviations from three independent experiments

The selected media components were used in *T. trachyspermus* NBRC 32238 culture, to prove the effectiveness of SA production. This strain was cultivated in a flask with three baffles containing 100 g/L glucose at 28 °C and 140 rpm for 9 days. The nitrogen source and trace metal compound used were 4.5 g/L meat extract and 5.0 g/L FeCl_3_. Glucose consumption started from day 3 and was almost depleted at day 7, and correspondingly, the cells grew with the progress of culture and reached a DCW of 12.7 g/L at day 7 (Fig. 9a). The maximum amount of SA was 29.0 g/L, which was 2.4-fold higher than that of the basic medium culture. The SA yield of 29% on this strain is close to that of 35% on the industrial strain of *P. spiculisporum* Lehman No.10-1 (Tabuchi et al. 1977).

In terms of pH behavior, SA production started after the pH fell below 2.4, at day 3. The produced SA decreased after a maximum amount, either because the strain assimilated the product post-glucose decrease, or because it dissolved in the produced ethanol, to be filtered through the filter paper. However, because the product is crystalline in low pH conditions, the reason for this phenomenon is unclear. From the results of the flask batch culture, the growth of the mycelium cells became constant after 4 days because of the decrease in the pH of the medium. SA production stopped with glucose depletion on day 7. A scale-up of batch equipment is necessary to make the process continuous and to produce the product stably and efficiently.

A bioreactor culture of *T. trachyspermus* NBRC 32238 was performed in a 1-L working volume with 100 g/L glucose. The culture was carried out for 11 days at 28 °C and 700 rpm with an aeration rate of 0.5 vvm. The strain assimilated the substrate for 7 days and the DCW was almost constant at approximately 11.2 g/L after 10 days (Fig. 9b). SA production started on day 4 and reached a value of 25.3 g/L at day 8, which was lower than that in the flask culture. It also decreased after glucose depletion, similar to that in the flask culture. In the glucose culture using both flask and bioreactor, the strain requires 3–4 days to decrease the pH to 2.4 and start producing SA. Scale-up of the aerobic processes often reduces the biomass yield and production of the target substance. The use of filamentous fungi is particularly problematic because of the entanglement of hyphae with the equipment. Dispersion is an important factor for substrates, cells, and oxygen supply in bioprocesses using aerobic microorganisms. Optimization of these conditions improves the production of the target substance.

### SA production using fed-batch culture

One of the major causes of decreased SA production was substrate depletion in the batch culture. A fed-culture was performed to improve the SA production, with the stepwise supply of an additional medium containing glucose to the bioreactor. The productivity of SA was improved upon supplying sufficient oxygen and a carbon source to the cells.

**Fig. 10:**
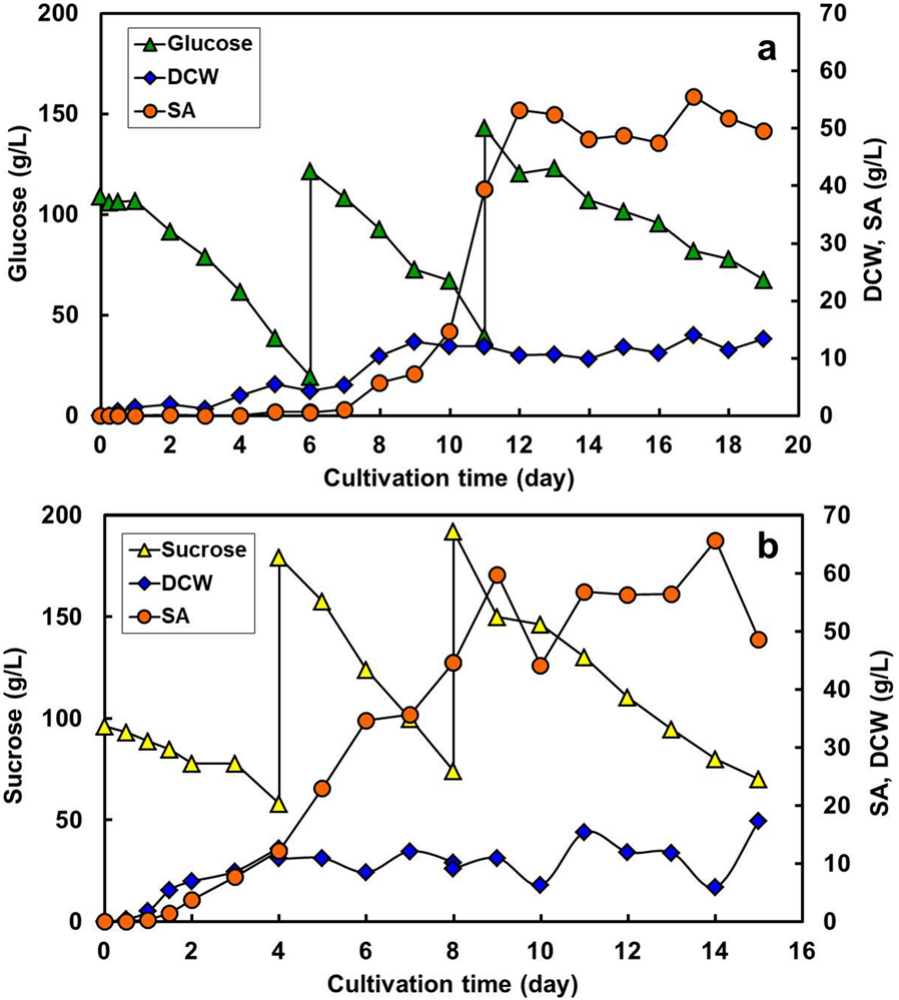
Fed-batch culture for spiculisporic acid production from different carbon source by *T*. *trachyspermus* NBRC 32238. **a** Glucose culture, with feeding of additional sugar, at 6 and 12 days. **b** Sucrose culture, with feeding of additional sugar, at 4 and 8 days. The cultures were contained 4.5 g/L meat extract, and 5.0 g/L FeCl_3_ and operated in an aeration stirring-type reactor containing 1 L medium controlled at 28 °C and 700 rpm with an aeration rate of 0.5 vvm for 19 days in case of glucose culture and for 15 days in case of sucrose culture

A fed-batch culture was performed with *T. trachyspermus* NBRC 32238 and glucose. Fermentation was first started with 100 g/L of substrate, 4.5 g/L meat extract, and 5.0 g/L FeCl_3_, under the same conditions as the batch culture using the bioreactor. After incubation for 6 days, and then subsequently, again at the interval of 6 days, 200 mL of 500 g/L glucose was added directly to the bioreactor at the point at which SA production stagnates. The residual glucose concentration decreased to 19.5 g/L and the cell grew to a DCW of 4.3 g/L in the early 6 days of cultivation (Fig. 10a). At the same time, the produced SA was only 0.66 g/L and hardly increased, although the strain was growing by consuming glucose in the culture. After the addition of glucose, the sugar gradually decreased at the same rate as the first batch term, with increasing DCW; in addition, SA production increased drastically with growth and reached 39.5 g/L on day 11. It further increased to 53.2 g/L on day 12, but still the DCW hardly increased after glucose was added again, because the mycelia amount in the reactor was limited. The productivity of the fed-batch culture was 4.1 g/L/day on day 12. It was constant at 47–55 g/L from day 12 to 19; however, glucose was slowly consumed for maintenance of the mycelia. The product reached its limit because of the spatial inhibition in the reactor and other biochemical reasons of the strain, such as product inhibition and toxicity. SA also did not decrease as in the batch culture because adequate sugar remained around the mycelium in the fed-culture. The strain can consume sufficient sugar in this culture in the late term, while the depletion of glucose results in SA consumption in the batch culture.

Sucrose fed-batch culture was attempted using stepwise addition with *T. trachyspermus* NBRC 32238, as the strain can produce SA from sucrose. The culture was performed using the same equipment and conditions, with the replacement of glucose with sucrose at a concentration of 100 g/L. The substrate was fed on days 4 and 8, which were the timings of growth and stagnation of SA production, respectively. The residual sucrose decreased to 58 g/L with the growth of the cells to a DCW of 12.6 g/L at day 4 (Fig. 10b). After the two additions, the growth of the mycelium remained almost constant from day 4 until the end of the culture (15 days). The growth of the strain was promoted by sucrose, as evidenced by the observation that the maximum growth was 5 days faster than that with glucose. The SA yield was 12.1 g/L in the sucrose culture at only day 4, although it was hardly produced in the early term with glucose. Only 42 g/L of sucrose consumption resulted in a DCW of 12.6 g/L and an SA amount of 12.1 g/L. After the first addition, sucrose was rapidly consumed, from 179 g/L to 74 g/L, and simultaneously, SA amount increased to 44.7 g/L from day 4 to day 8 of the culture. Upon adding the sucrose solution again on the 8th day, the SA yield reached 59.7 g/L and remained almost constant (56–60 g/L). Sucrose was consumed only to maintain the cells. The productivity of the sucrose fed-batch culture was 6.6 g/L/day on day 9, which was 1.6-fold higher than that of the glucose culture. The SA production was lower than that of *P. spiculisporum* which produced 110 g/L from total glucose of 160 g/L by 4 times feeding for 11 days (Tabuchi et al. 1977). However, the rapid growth and SA production indicated that sucrose is a better carbon source than glucose in fed-batch SA production. Using sucrose for the manufacture of a compound has the advantage that raw materials are easily available, which keeps the unit cost relatively low. A further increase in SA production is expected upon carrying out continuous culture, which involves partially extracting the culture solution or sedimentation and separation of the product during the culture.

The fed-batch culture process is usually a step in an R and D program that leads to large-scale industrial production by fermentation. The operation of a fed-batch system for improving the productivity of a product is often governed by a number of important engineering considerations, such as improving the culture equipment and conditions. This process has the following effects: (1) decreases the viscosity of the culture solution; (2) increases the density of the cells; (3) compensates for water loss; (4) extends the production time; (5) suppresses the Crabtree effect for high-concentration production; (6) suppresses catabolite repression in secondary metabolite production; and (7) controls the expression of genes with repressible promoters. Fed-batch culture is desirable for a variety of industries that deal with living organisms, such as the production of secondary metabolites, large-scale chemicals, and pharmaceuticals that are produced by overexpressing recombinant or natural strains (Elsayed et al. 2015; Krause et al. 2016).

## Conclusion

In the present study, we found that among 11 related strains of *Talaromyces*, the novel ascomycetes *T. trachyspermus* NBRC 32238 had the highest production capability for SA, a biosurfactant with multivalent carboxyl groups. The strain can produce SA effectively from glucose and sucrose at low pH (no more than 3.0). Low pH culture is an advantage that avoids contamination in bioprocesses using microorganisms. Nitrogen sources and metals in the medium affect the production of secondary metabolites, including glycolytic and metabolic pathways, redox reactions, and secretion of enzymes. The SA yield was improved upon in the sequential identification of an optimum nitrogen source and trace metal component in the medium. Meat extract and FeCl_3_ were found to be the appropriate components for this production. The factor of a positive interaction becomes clear by examining the detailed composition of the components and the metabolism when they are added. A typical problem faced in the scale-up of fermentation processes is that even though the cells continue to grow, the products hardly appear. Reactor culture is different from that carried out in a flask, in terms of aeration, distribution, shear force, etc*.* The reactor culture of SA production reproduced the flask culture at the same level. In this experimental system, the strain used the substrate for simultaneous cell growth and SA production. To improve the yield, fed-batch fermentation was applied as a continuous production system. Periodic addition of substrate increased the final SA amount and prevented product consumption. Sucrose culture enhanced SA production by *T. trachyspermus*. A remarkable SA of 60 g/L was achieved using the fed-batch process. The fed-batch strategy is a promising, time-saving strategy from a long-term perspective, because the process can produce a high amount of substance in one tank. SA is expected to be widely used in cosmetics, food, medical materials, etc. In the near future, we hope that industrially produced SA using these strains is of application in many fields.

## Data Availability

The dataset (graphs and tables) supporting the conclusions of this article is available.

## References

[CR1] Ahmadi-Ashtiani HR, Baldisserotto A, Cesa E (2020). Microbial biosurfactants as key multifunctional ingredients for sustainable cosmetics. Cosmetics.

[CR2] Banat IM, Makkar RS, Cameotra SS (2000). Potential commercial applications of microbial surfactants. Appl Microbiol Biotechnol.

[CR3] Benjamin CR (1955). Ascocarps of *Aspergillus* and *Penicillium*. Mycologia.

[CR4] Brandänge S, Dahlman O, Lindqvist B (1984). Absolute configuration and enantioselective synthesis of spiculisporic acid. Acta Chem Scand.

[CR5] Brown SP, Goodwin NC, MacMillan DWC (2003). The first enantioselective organocatalytic Mukaiyama–Michael reaction: a direct method for the synthesis of enantioenriched γ-butenolide architecture. J Am Chem Soc.

[CR6] Chakravarty I, Kundu K, Ojha S (2017). Development of various processing strategies for new generation antibiotics using different modes of bioreactors. JSM Biotechnol Bioeng.

[CR7] Chen WC, Juang RS, Wei YH (2015). Applications of a lipopeptide biosurfactant, surfactin, produced by microorganisms. Biochem Eng J.

[CR8] Choi YK, Lee CH, Takizawa Y (1993). Tricarboxylic acid biosurfactant derived from spiculisporic acid. J Jpn Oil Chem Soc.

[CR9] Clutterbuc PW, Raistrick H, Rintoul ML (1931). On the production from glucose by *Penicillium spiculisporum* lehman of a new polybasic fatty acid, C_17_H_2_8O_6_ (The lactone of γ -hydroxy-β δ -dicarboxypentadecoic acid). Philos Trans R Soc London Ser Contain Pap Biol Character.

[CR10] De Almeida DG, de Soares Da Silva R, Luna JM (2016). Biosurfactants: promising molecules for petroleum biotechnology advances. Front Microbiol.

[CR11] El Moslamy SH (2019). Application of fed-batch fermentation modes for industrial bioprocess development of microbial behaviour. Ann Biotechnol Bioeng.

[CR12] Elazzazy AM, Abdelmoneim TS, Almaghrabi OA (2015). Isolation and characterization of biosurfactant production under extreme environmental conditions by alkali-halo-thermophilic bacteria from Saudi Arabia. Saud J Biol Sci.

[CR13] Elsayed EA, Omar HG, El-Enshasy HA (2015). Development of fed-batch cultivation strategy for efficient oxytetracycline production by *Streptomyces rimosus* at semi-industrial scale. Brazilian Arch Biol Technol.

[CR14] Fennell DI (1973). The Genus *Talaromyces*: studies on *Talaromyces* and related genera II by Amelia C. Stolk, R. A. Samson. Mycologia.

[CR15] Frumkin H, Hess J, Vindigni S (2009). Energy and public health: the challenge of peak petroleum. Public Health Rep.

[CR16] Hong JJ, Yang SM, Lee CH (1998). Ultrafiltration of divalent metal cations from aqueous solution using polycarboxylic acid type biosurfactant. J Colloid Interface Sci.

[CR17] Ishigami Y, Zhang Y, Ji F (2000). Spiculisporic acid. functional development of biosurfactant. Chim Oggi-Chem Today.

[CR18] Ishigami Y, Zhang Y, Goto M (2013). Molecular and crystal structure of spiculisporic acid and correlation with the surface activity. J Oleo Sci.

[CR19] Krause M, Neubauer A, Neubauer P (2016). The fed-batch principle for the molecular biology lab: controlled nutrient diets in ready-made media improve production of recombinant proteins in *Escherichia*
*coli*. Microb Cell Fact.

[CR20] Kumla D, Dethoup T, Buttachon S (2014). Spiculisporic acid E, a new spiculisporic acid derivative and ergosterol derivatives from the marine-sponge associated fungus *Talaromyces trachyspermus* (KUFA 0021). Nat Prod Commun.

[CR21] Kunieda H, Sakamoto K (2010). Function and application of surfactant and amphiphilic polymers.

[CR22] Mahamallik P, Pal A (2017). Degradation of textile wastewater by modified photo-Fenton process: application of Co(II) adsorbed surfactant-modified alumina as heterogeneous catalyst. J Environ Chem Eng.

[CR23] Måhlén A (1971). Properties of 2-decylcitrate synthase from *Penicillium spiculisporum* lehman. Eur J Biochem.

[CR24] Måhlén A (1973). Purification and some properties of 2-decylhomocitrate synthase from *Penicillium spiculisporum*. Eur J Biochem.

[CR25] Melo RPF, Barros Neto EL, Moura MCPA (2015). Removal of direct yellow 27 dye using animal fat and vegetable oil-based surfactant. J Water Process Eng.

[CR26] Pekdemir T, Tokunaga S, Ishigami Y, Hong KJ (2000). Removal of cadmium or lead from polluted water by biological amphiphiles. J Surfactants Deterg.

[CR27] Raffa P, Wever DAZ, Picchioni F, Broekhuis AA (2015). Polymeric surfactants: Synthesis, properties, and links to applications. Chem Rev.

[CR28] Rufino RD, de Luna JM, de Campos Takaki GM, Sarubbo LA (2014). Characterization and properties of the biosurfactant produced by *Candida lipolytica* UCP 0988. Electron J Biotechnol.

[CR29] Santos DKF, Rufino RD, Luna JM (2016). Biosurfactants: multifunctional biomolecules of the 21st century. Int J Mol Sci.

[CR30] Shiozawa H, Takahashi M, Takatsu T (1995). Trachyspic acid, a new metabolite produced by *Talaromyces* trachyspermus, that inhibits tumor cell heparanase: taxonomy of the producing strain, fermentation, isolation, structural elucidation, and biological activity. J Antibiot (tokyo).

[CR31] Soberón-Chávez G, Lépine F, Déziel E (2005). Production of rhamnolipids by *Pseudomonas aeruginosa*. Appl Microbiol Biotechnol.

[CR32] Stolk AC, Samson RA (1971). Studies on *Talaromyces* and related genera I. *Hamigera* gen. nov. and *Byssochlamys*. Persoonia-Mol Phylogeny Evol Fungi.

[CR33] Tabuchi T, Isei N, Etsuko H, Kobayashi H (1977). Factors affecting the production of the open-ring acid of spiculisporic acid by *Penicillium spiculisporum*. J Ferment Technol.

[CR34] Varjani SJ, Upasani VN (2017). Critical review on biosurfactant analysis, purification and characterization using rhamnolipid as a model biosurfactant. Bioresour Technol.

[CR35] Vijayakumar S, Saravanan V (2015). Biosurfactants—types, sources and applications. Res J Microbiol.

[CR36] Wang R, Liu TM, Shen MH (2012). Spiculisporic acids B-D, three new γ-butenolide derivatives from a sea urchin-derived fungus *Aspergillus* sp. HDf2. Molecules.

[CR37] Wang R, Guo ZK, Li XM (2015). Spiculisporic acid analogues of the marine-derived fungus, *Aspergillus candidus* strain HDf2, and their antibacterial activity. Antonie Van Leeuwenhoek Int J Gen Mol Microbiol.

[CR38] Yamazaki S, Ishigami Y, Gama Y (1983). Emulsion copolymerization of ethyl acrylate and methyl methacrylate by using biosoap with polyfunctional groups. Kobunshi Ronbunshu.

[CR39] Year Book of Current Production Statics, Chemical Industry (2019) Ministry of Economy, Trade and Industry, Japan. https://www.meti.go.jp/statistics/tyo/seidou/result/gaiyo/resourceData/02_kagaku/nenpo/h2dbb2019k.pdf. Accessed 05 Mar 2021

